# Silver Nanoparticles of *Artemisia sieberi* Extracts: Chemical Composition and Antimicrobial Activities

**DOI:** 10.3390/plants12112093

**Published:** 2023-05-24

**Authors:** Fatimah Al-Otibi, Nourah A. Alshammry, Raedah I. Alharbi, May N. Bin-Jumah, Maha M. AlSubaie

**Affiliations:** 1 Department of Botany and Microbiology, College of Science, King Saud University, P.O. Box 22452, Riyadh 11495, Saudi Arabia; raalharbi@ksu.edu.sa (R.I.A.); 441203885@student.ksu.edu.sa (M.M.A.); 2 Department of Biology, College of Science, Health Science Research Center, Princess Nourah Bint Abdulrahman University, Riyadh 11474, Saudi Arabia; nourah.khalawi@gmail.com (N.A.A.); mnbinjumah@pnu.edu.sa (M.N.B.-J.); 3 Environment and Biomaterial Unit, Health Sciences Research Center, Princess Nourah Bint Abdulrahman University, Riyadh 11474, Saudi Arabia; 4 Saudi Society for Applied Science, Princess Nourah Bint Abdulrahman University, Riyadh 11474, Saudi Arabia

**Keywords:** antifungal, antibacterial, *Artemisia sieberi*, silver nanoparticles, pathogenic microbes, TEM analysis

## Abstract

Background: *Artemisia sieberi* (mugwort) is a member of the daisy family Asteraceae and is widely propagated in Saudi Arabia. *A. sieberi* has historical medical importance in traditional societies. The current study aimed to assess the antibacterial and antifungal characteristics of the aqueous and ethanolic extracts of *A. sieberi*. In addition, the study investigated the effect of silver nanoparticles (AgNPs) synthesized from the *A. sieberi* extract. Methods: The ethanolic and aqueous extracts and AgNPs were prepared from the shoots of *A. sieberi*. The characteristics of AgNPs were assessed by UV–visible spectroscopy, transmission electron microscopy (TEM), Fourier transform infrared spectroscopy (FTIR), and dynamic light scattering (DLS). The antibacterial experiments were performed against *Staphylococcus aureus*, *Bacillus subtilis*, *Escherichia coli*, and *Pseudomonas aeruginosa*. The fungal species used were *Candida parapsilosis*, *Candida krusei*, *Candida famata*, *Candida rhodotorula*, and *Candida albicans*. The antibacterial and antifungal characteristics were evaluated by measuring the diameter of growing organisms in Petri dishes treated with different concentrations of either extracts or AgNPs compared to the untreated controls. Furthermore, TEM imaging was used to investigate any ultrastructure changes in the microbes treated with crude extracts and AgNO_3_. Results: The ethanolic and aqueous extracts significantly decreased the growth of *E. coli*, *S. aureus*, and *B. subtilis* (*p* < 0.001), while *P. aeruginosa* was not affected. Unlike crude extracts, AgNPs had more substantial antibacterial effects against all species. In addition, the mycelial growth of *C. famata* was reduced by the treatment of both extracts. *C. krusei* mycelial growth was decreased by the aqueous extract, while the growth of *C. parapsilosis* was affected by the ethanolic extract and AgNPs (*p* < 0.001). None of the treatments affected the growth of *C. albicans* or *C. rhodotorula*. TEM analysis showed cellular ultrastructure changes in the treated *S. aureus* and *C. famata* compared to the control. Conclusion: The biosynthesized AgNPs and extracts of *A. sieberi* have a potential antimicrobial characteristic against pathogenic bacterial and fungal strains and nullified resistance behavior.

## 1. Introduction

*Artemisia* spp. is an important imperishable shrubby medicinal herb among the 500 different species related to the Asteraceae family. It is an imperishable plant native to Asia, the Middle East, Europe, and North Africa [1]. *Artemisia* spp. has several conversational names including green gusto, absinthe, absinthium, and mugwort. The flower heads are short, nearly orbicular, and hang in a standing, lush panicle, and the little flowers are pendulum-like with a greenish-unheroic color. The leaves and flowers are veritably bitter, with a distinctive aroma suggesting that of thujone [2].

Essential oils uprooted from *Artemisia* spp. have a broad spectrum of bioactivity due to the presence of several active constituents or secondary metabolites [3]. Extraction processes, similar to the distillation of aromatic compounds, yielded sufficient essential oils containing a variety of unpredictable molecules such as terpenes, phenolic-derived aromatics, and aliphatic factors [4]. In addition, the low cellular toxicity of the extracted herbal active constituents suggested it as a sustainable antimicrobial, antiviral, and anticancer medicine with fewer side effects.

Recent reports revealed that the uprooted essential oils from *Artemisia* spp. and other active factors have antibacterial, antifungal, and antiviral properties [2,5]. It was reviewed that bitter sesquiterpenoid lactones, flavonoids, and other bitterness-conducting composites, azulenes, phenolic acids, tannins, and lignans are the main substances responsible for the natural exertion of the *Artemisia* spp. [6,7]. Lately, antiprotozoal, antibacterial, antifungal, anti-ulcer, hepatoprotective, anti-inflammatory, immunomodulatory, cytotoxic, analgesic, neuroprotective, anti-depressant, precognitive, neurotrophic, cell membrane stabilizing, and antioxidant conditioning effects emerged as multitudinous other directions of natural exertion of the factors of *Artemisia* spp. [6,8,9]. Therefore, biomaterials with high antimicrobial, antiviral, and antioxidant exertion can replace antibiotic medicines [10].

*Artemisia sieberi* grows hectically in the Tabuk region, the northwestern part of Saudi Arabia. Numerous times, *A. sieberi*, as a traditional medicinal condiment, has enjoyed a reputation among condiment experts in Arabian countries such as Egypt and Saudi Arabia. In Saudi Arabia, the Tabuk region is characterized by largely variable environmental conditions with temperatures ranging from extremely low to high, which are considerable for growth and variations in *A. sieberi* [11]. A former study revealed that the unpredictable extracts of *A. sieberi* are composed of cis-davanone, camphor, terpinol, E-nerolidol, and linalool, which have significant antimicrobial conditioning against different bacterial and fungal species [3].

Nanotechnology has emerged as a new, rapidly developing study topic with several applications in recent years. With growing environmental concerns, employing green methods for nanomaterial production is a significant problem [12]. Green, environmentally friendly procedures in chemistry and chemical technologies are becoming increasingly popular and are desperately needed due to global environmental challenges [13]. Because of their appealing physiochemical features, silver nanoparticles (AgNPs) play a significant role in biology and medicine. Silver compounds have long been recognized to have potent inhibitory and bactericidal effects, as well as a wide range of antimicrobial properties, and have been used for millennia to prevent and cure various ailments, most notably infections [14]. The literature has stated that nanostructured systems based on nanotechnology can improve the characteristics of plant extracts. This might enhance several extract features, such as plant extract action, increase the sustained release of active ingredients, lower the needed dose, lessen side effects, and improve activity [15]. 

Several studies have used nanostructured devices to improve the characteristics of plant extracts. In the studies conducted by Huq in 2020, biosynthesized AgNPs from *Lysinibacillus xylanilyticus* and *Paenibacillus* spp. strains showed considerable antibacterial activity against *Vibrio parahaemolyticus* and *Salmonella Typhimurium* [16,17]. Other research employed lipid-based systems to combine a combination of green tea and ginseng extracts in different formulations to boost active component absorption [18]. In addition, liposome and nanoparticle technology were used to improve and help the active components of *Artemisia arborescens* L. (Asteraceae) penetrate the cytoplasmic viral barrier [19], and methanolic extracts of *Ocimum sanctum* L. (*Lamiaceae*) improved the encapsulation of the extract to provide better antimicrobial activity than in free-form preparations [20]. Using different nanotechnology-based drug delivery systems, such as polymeric nanoparticles, solid lipid nanoparticles, liquid crystal systems, and precursor systems for liquid crystals, liposomes, and microemulsions, is an intriguing approach to improving the most desirable properties of a formulation [21].

The current study aimed to estimate the use of silver nanoparticles (AgNPs) synthesized from the aerial parts (shoot) of the *A. sieberi* strain growing in the northern region of Saudi Arabia as an antimicrobial agent against different bacterial and fungal species. To our knowledge, this is the first research to investigate the antimicrobial activities of ethanolic and aqueous extracts of the shoots of *A. sieberi* against specific pathogens, as most of the previous studies reported these activities from essential oils only. 

## 2. Results

### 2.1. Chemical Composition of A. sieberi

**Figure 1:**
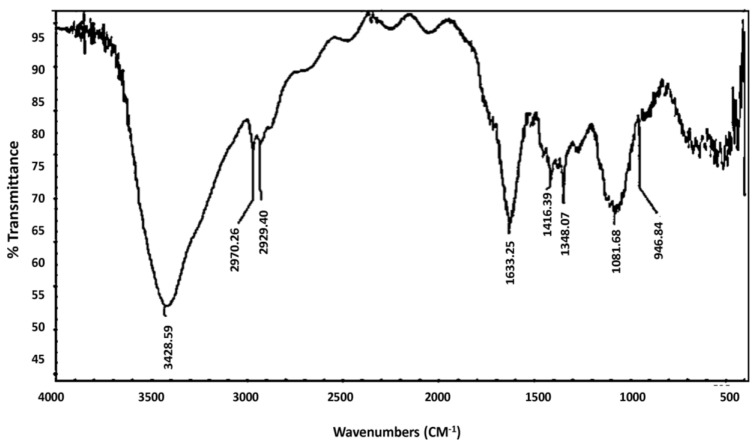
FTIR results of the aqueous extract of *A. sieberi*. The results were produced by the Nicolet 6700 FTIR Spectrometer at 500–4000/cm.

**Figure 2:**
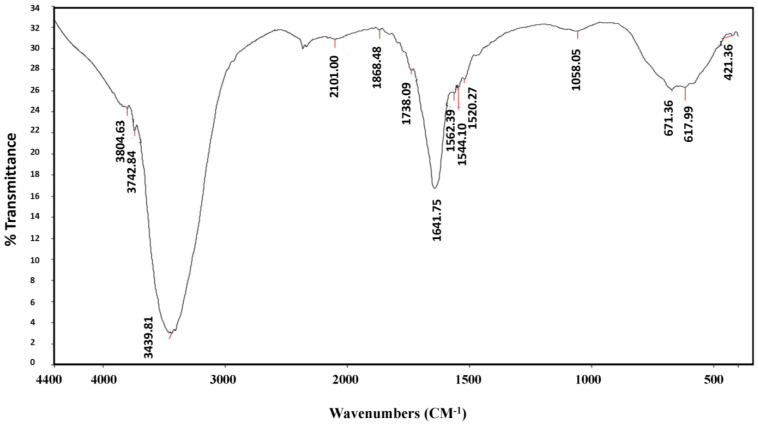
FTIR results of AgNPs biosynthesized from the aqueous extract of *A. sieberi*. The results were produced by the Nicolet 6700 FTIR Spectrometer at 500–4000/cm.

The aqueous extract and the biosynthesized AgNPs of *A. sieberi* were analyzed by FTIR to identify the important functional groups. As shown in Figure 1 and Figure 2, the FTIR spectrum confirmed the presence of various functional groups.

The aqueous extract of *A. sieberi* was found to be rich in amine salts (N-H), amines (C-N), alcohols (O-H), and alkenes (C=C), as shown in Table 1. Biosynthesized AgNPs were rich in alkynes (CΞC), alcohols (O-H), alkenes (C=C), aromatic (C-H), and nitro compounds (N-O). 

The chemical composition of the ethanolic extract of *A. sieberi* was phytochemically examined by GC-MS analysis (Table 2). The GC-MS results confirmed the presence of multiple biomolecules, including ethylene glycol surfactants (2-hexoxyethanol and chloral), ethers (dihexyl ether), volatile fatty acids (dichloroacetic acid), alkanes (piperidine), alkane sulfonic acids (N, N-Bis(2-hydroxyethyl)-2-aminoethanesulfonic acid), and aromatic compounds (phenprobamate, cumene hydroperoxide, and 4-ethyl-o-xylene).

### 2.2. Biosynthesis and Characterization of A. sieberi AgNPs

**Figure 3:**
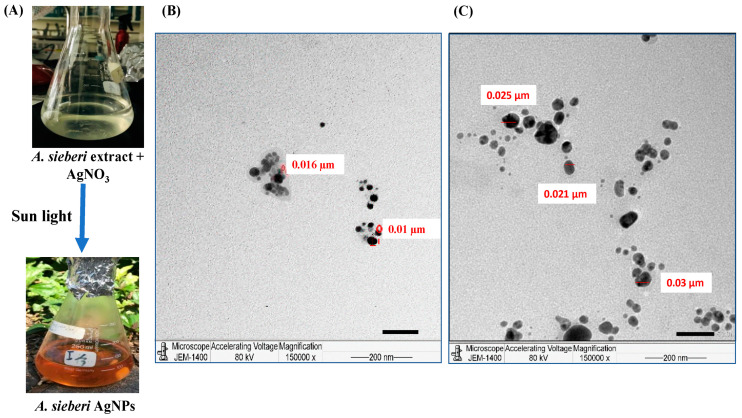
Characterization of the synthesized *A. sieberi* AgNPs by the JEOL JEM-1400 transmission electron microscope. (**A**) The synthesis process of AgNPs from the aqueous extract *A. sieberi* and AgNO_3_ incubated in sunlight and caused the color change from colorless to brown. (**B**,**C**) TEM micrographs of (**B**) *A. sieberi* extract and (**C**) *A. sieberi* AgNPs, in which the shape and size are shown in µm.

AgNPs were biosynthesized from the aqueous extract of *A. sieberi*. The synthesis process of AgNPs was interrupted by the change in color of AgNO_3_ (2 µM) mixed with the extract of *A. sieberi* (20%) from colorless to brown (Figure 3A). The transmission electron microscopy (TEM) images confirmed the biosynthesis of AgNPs, which appeared in a spherical shape with a diameter size of 27.5 ± 3.5 nm (Figure 3B) compared to the 13 ± 2.42 nm of the unconjugated crude extract particles (Figure 3C). 

**Figure 4:**
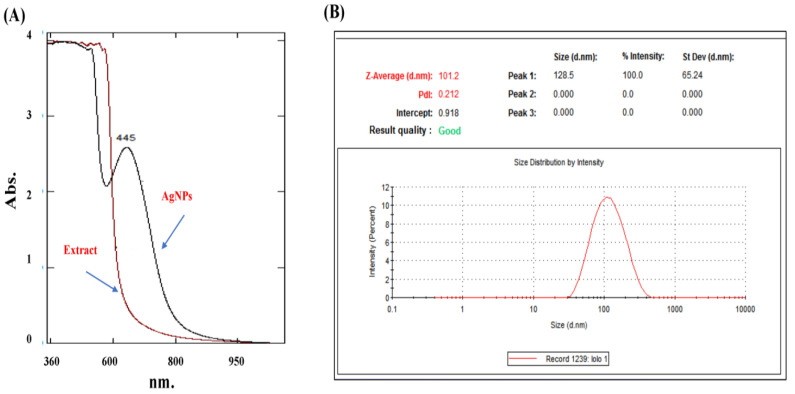
Characterization of the synthesized *A. sieberi* AgNPs. (**A**) The UV–visible spectrum of *A. sieberi* extract and AgNPs; the spectrum was analyzed by the Shimadzu UV–visible spectrophotometer. (**B**) TEM micrograph of *A. sieberi* extract in which the shape and size are shown in µm.

The purified AgNPs were analyzed by UV–visible spectra (Figure 4A), which showed a surface plasmon resonance (SPR) peak at 445 nm. The results were compared to the spectrum obtained by the aqueous extract of *A. sieberi*, which revealed a comparable lower spectrum than that of the biosynthesized AgNPs. Furthermore, to measure the stability of the colloidal nanoparticles, we measured their effective surface charge, or zeta potential, which reflected their surface energy. The dynamic light scattering (DLS) method calculated the polydispersity index (PDI) (0.212) and the Z-average (101.2 d.nm) of AgNPs (Figure 4B). 

### 2.3. Antibacterial Activities of A. sieberi

**Figure 5:**
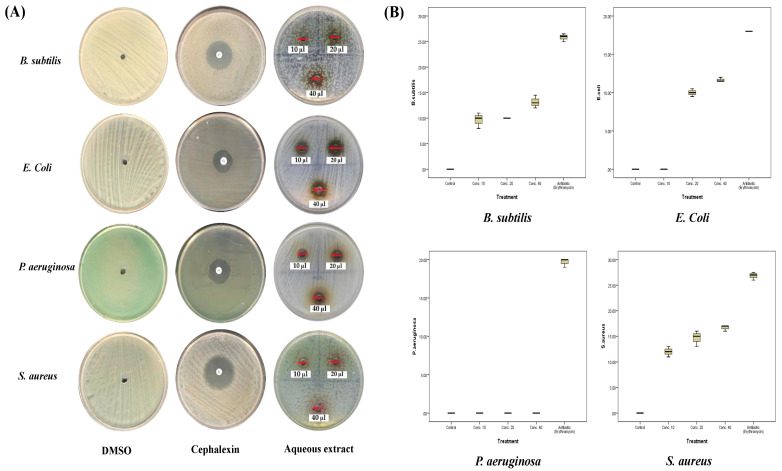
Antibacterial activities of the aqueous extract of *A. sieberi*. The growth inhibition zone was measured by the well diffusion method for the species growing on the Mueller–Hinton agar dishes. (**A**) Petri dishes of different bacterial strains treated with either DMSO (negative control, <1%), cephalexin disc (positive control, 5 µg/mL), or different amounts of the aqueous extract of *A. sieberi* (10, 20, and 40%). (**B**) Box plots showed the antibacterial effect of different concentrations of the aqueous extract of *A. sieberi* compared to DMSO and cephalexin.

In the current study, three doses of both extracts of *A. sieberi* were used to assess their antibacterial activities on the growth of different species (*S. aureus*, *P. aeruginosa*, *B. subtilis*, and *E. coli*) and compare them to antibacterial effects of the cephalexin, a known broad-spectrum antibiotic, at a dose of 5 µg/mL. The zone of inhibition of each treatment was measured in mm and compared to the negative control of DMSO-treated species (1 µL/dish). At the doses of 20 µg/mL and 40 µg/mL of the aqueous extract of *A. sieberi* (Figure 5), significant inhibition of the growth of *S. aureus*, *B. subtilis*, and *E. coli* (*p* < 0.001) was shown, while the growth of *P. aeruginosa* was not affected. As shown in Table 3 and Figure 5, the largest inhibition zones were for *S. aureus* (3, 16.7 ± 0.6 mm, MIC: <10 µg/mL), followed by *B. subtilis* (13.2 ± 1.3 mm, MIC: <10 µg/mL) and *E. coli* (11.7 ± 0.3 mm, MIC: 15 µg/mL) at the dose of 40 µg/mL. The box-plot analysis represents the medians (solid horizontal lines) and the means (the square box) of each zone of inhibition (ZOI) of each strain. As shown in Figure 5B, the box plots showed low median ranges and a standard deviation (SD) among all replicates, which indicated accurate and constant imbibitional effects. 

**Figure 6:**
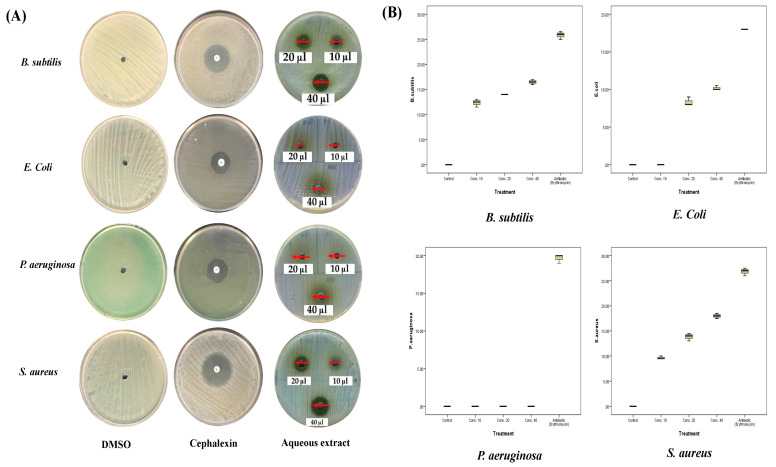
Antibacterial activities of the ethanolic extract of *A. sieberi*. The growth inhibition zone was measured by the well diffusion method for the species growing on the Mueller–Hinton agar dishes. (**A**) Petri dishes of different bacterial strains treated with either DMSO (negative control, 1 µL), cephalexin disc (positive control, 5 µg/mL), or different amounts of the ethanolic extract of *A. sieberi* (1 mg/mL). (**B**) Box plots showed the antibacterial effect of different concentrations of the ethanolic extract of *A. sieberi* compared to DMSO and cephalexin.

Similarly, different doses of the ethanolic extract of *A. sieberi* (Figure 6) showed significant inhibition of the growth of *S. aureus*, *B. subtilis*, and *E. coli* (*p* < 0.001), while the growth of *P. aeruginosa* was not affected. As shown in Table 3 and Figure 4, the largest inhibition zones were for *S. aureus* (18 ± 0.5 mm, MIC: <10 µg/mL), followed by *B. subtilis* (16.5 ± 0.5 mm, MIC: <10 µg/mL) and *E. coli* (10.2 ± 0.3 mm, MIC: 15 µg/mL) at the dose of 40 µg/mL. Comparing both results, it was obvious that the ethanolic extract had more inhibitory effects than the aqueous extract against *S. aureus* (median: 18 versus 17 mm) and *B. subtilis* (median: 16.5 versus 13 mm). In contrast, *E. coli* growth was more sensitive to the aqueous (median: 11.5 mm) than the ethanolic extract (median: 10 mm) (Table 3). In addition, the box plots showed low median ranges and SD among all replicates, which indicated accurate and constant imbibitional effects.

**Figure 7:**
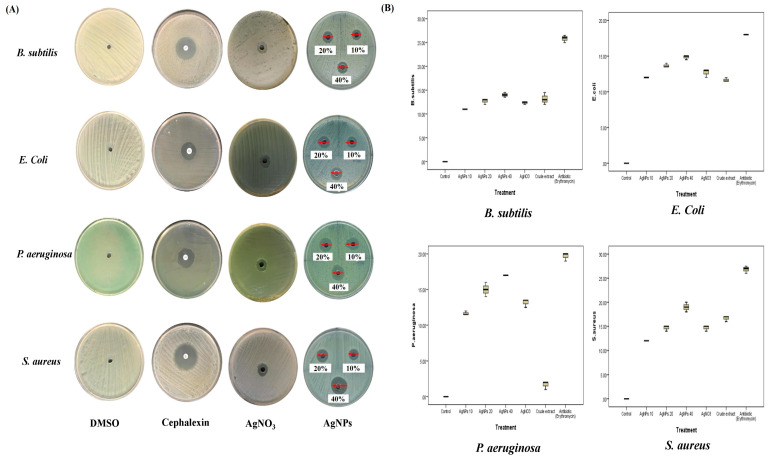
Antibacterial activities of biosynthesized AgNPs of *A. sieberi*. The growth inhibition zone was measured by the well diffusion method for the species growing on the Mueller–Hinton agar dishes. (**A**) Petri dishes of different bacterial strains treated with either DMSO (negative control, 1 µL), cephalexin disc (positive control, 5 µg/mL), AgNO_3_ (2 mM, 20% *v/v*), or different amounts of AgNPs. (**B**) Box plots showed the antibacterial effect of different concentrations of the AgNPs compared to DMSO, cephalexin, and AgNO_3_ (2 mM).

The biosynthesized AgNPs induced significant inhibitory effects against all strains at all concentrations (*p* < 0.001), as shown in Table 3 and Figure 7. All calculated MIC results were <10 µg/mL for AgNPs, which indicated stronger effects. The largest inhibition zones were for *S. aureus* (19 ± 1 mm), followed by *P. aeruginosa* (17 ± 0.1 mm), *E. coli* (14.8 ± 0.3 mm), and *B. subtilis* (14 ± 0.5 mm) at the highest dose. Compared to the crude extract, AgNPs had the strongest inhibitory effects at all doses. The calculated ZOI of AgNO_3_ (2 mM) showed some inhibitory effect on the growth of all species, which was lower than AgNPs at all concentrations. In addition, the box plots showed low median ranges and SD among all replicates, which indicated accurate and constant inhibition effects. As shown from the above data, *S. aureus* was the most sensitive species to different treatments of *A. sieberi* among the other tested species. The comparative data analysis revealed that AgNPs induced higher zones of inhibition than the crude extracts. However, AgNPs induced significant growth inhibition of *B. subtilis* versus the ethanolic extract (*p* < 0.05) and *P. aeruginosa* versus both extracts *(p* < 0.001).

### 2.4. Antifungal Activities of A. sieberi

In the current study, three doses of both extracts of *A. sieberi* were used to assess their antifungal activities on the growth of different species (*C. rhodotorula*, *C. parapsilosis*, *C. krusei*, *C. famata*, and *C. albicans*). The results of both extracts were compared to a positive control of a known antifungal medicine (Terbinafine) at a dose of 5 µg/mL. 

**Figure 8:**
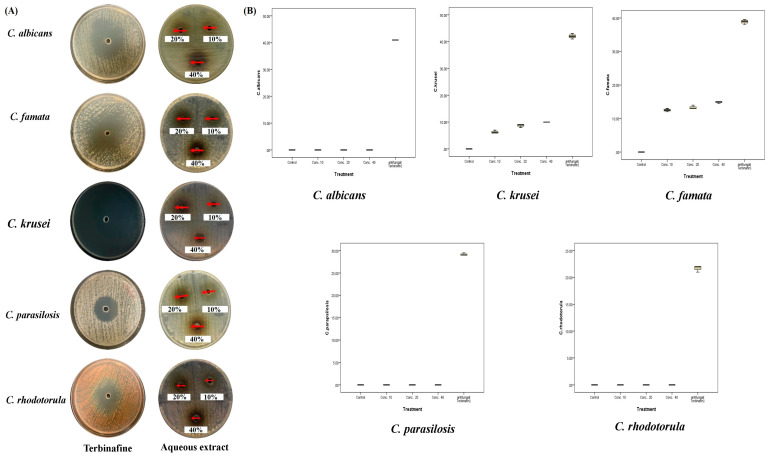
Antifungal activities of the aqueous extract of *A. sieberi*. The growth inhibition zone was measured by the well diffusion method for the species growing on the Sabouraud agar dishes. (**A**) Petri dishes of different fungal species treated with either terbinafine disc (positive control, 5 µg/mL) or different amounts of the aqueous extract of *A. sieberi* (10, 20, and 40%). (**B**) Box plots showed the antifungal effect of different concentrations of the aqueous extract of *A. sieberi* compared to terbinafine.

As shown in Figure 8 and Table 4, the aqueous extract of *A. sieberi* affected the mycelial growth of *C. krusei* and *C. famata* at all concentrations (*p* < 0.001). It was obvious that the greatest zone inhibition diameters were for *C. krusei* at the three doses. The mycelial growth of *C. rhodotorula*, *C. parapsilosis*, or *C. albicans* was not affected by any doses of the aqueous extract of *A. sieberi* (Figure 8). Despite variable effects, the terbinafine antifungal activity proved that all species were not restricted to any source of antifungal resistance. The box plot analysis revealed low SD and median values, which revealed the constant effect at all replicates. 

**Figure 9:**
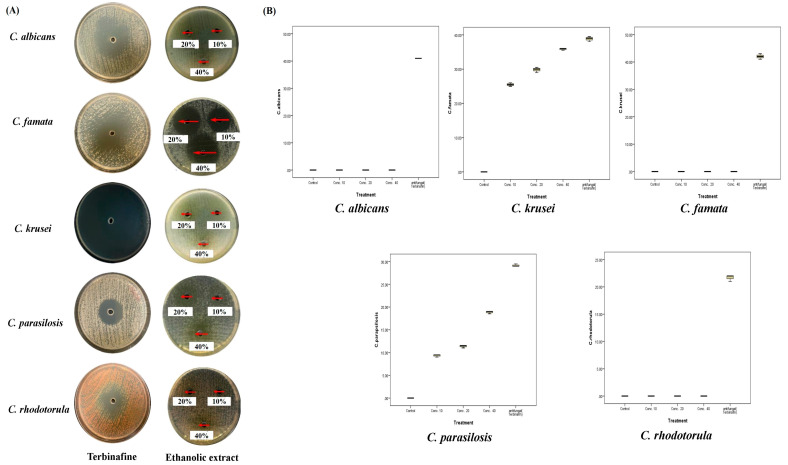
Antifungal activities of the ethanolic extract of *A. sieberi*. The growth inhibition zone was measured by the well diffusion method for the species growing on the Sabouraud agar dishes. (**A**) Petri dishes of different fungal species treated with either terbinafine disc (positive control, 5 µg/mL) or different amounts of the ethanolic extract of *A. sieberi* (10, 20, and 40%). (**B**) Box plots showed the antifungal effect of different concentrations of the ethanolic extract of *A. sieberi* compared to terbinafine.

For the ethanolic extract of *A. sieberi*, there was significant inhibition of the mycelial growth of *C. famata* and *C. parapsilosis* at all the concentrations (*p* < 0.001). Unlike the aqueous extract, the mycelial growth of *C. famata* treated with the ethanolic extract was the most affected (Figure 9). The mycelial growth of *C. rhodotorula*, *C. krusei*, or *C. albicans* was not affected by any doses (Figure 9). The box plot analysis revealed low SD and median values, which revealed the constant effect at all replicates. The highest inhibitory effect against *C. famata* was induced by the ethanolic extract (35.8 ± 0.3 mm) compared to the aqueous extract (14.8 ± 0.3 mm) at a dose of 40%. We noticed that the inhibitory effects of both plant extracts were higher than those of the AgNPs at different doses. However, AgNPs could inhibit the growth of all the tested *Candida* species (Table 4).

**Figure 10:**
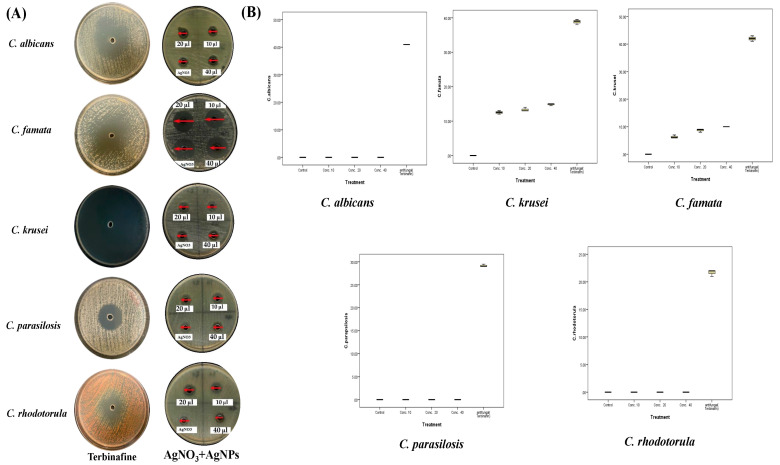
Antifungal activities of the biosynthesized AgNPs of *A. sieberi*. The growth inhibition zone was measured by the well diffusion method for the species growing on the Sabouraud agar dishes. (**A**) Petri dishes of different fungal species treated with either terbinafine disc (positive control, 5 µg/mL), AgNO3 (2 mM, 20% *v/v*), or different amounts of the ethanolic extract of *A. sieberi* (10, 20, and 40%). (**B**) Box plots showed the antifungal effect of different concentrations of the ethanolic extract of *A. sieberi* compared to terbinafine.

The effect of the biosynthesized AgNPs was stronger than that of both extracts of *A. sieberi* at all concentrations. Furthermore, AgNPs affected the mycelial growth of all the studied species (Figure 10). As shown in Table 4, the highest antifungal activity of AgNPs was against *C. famata* (26.2 ± 0.8 mm), followed by *C. krusei* (14.7 ± 0.8 mm), *C. albicans* (14.7 ± 0.8 mm), and *C. rhodotorula* (10.3 ± 0.6 mm), where the least sensitive species was *C. parapsilosis* (8.3 ± 0.6 mm) (Table 4). AgNO_3_ (2 mM) showed some inhibitory effect on the growth of all species, which was lower than that of AgNPs at all concentrations. In addition, the box plots showed low median ranges and SD among all replicates, which indicated accurate and constant inhibition effects. As shown from the above data, *C. famata* was the most sensitive species to different treatments of *A. sieberi* among the other tested species. The comparative data analysis revealed that the ethanolic extract induced more significant growth inhibition than the aqueous extract against *C. famata* and *C. parapsilosis* (*p* < 0.001). AgNPs induced higher zones of inhibition than the aqueous extract (*p* < 0.001). However, the ethanolic extract induced higher significant growth inhibition of *C. famata* and *C. parapsilosis* than AgNPs (*p* < 0.001).

### 2.5. Ultrastructural Characterizations by TEM of Selected Species Treated with Different Preparations of A. sieberi

**Figure 11:**
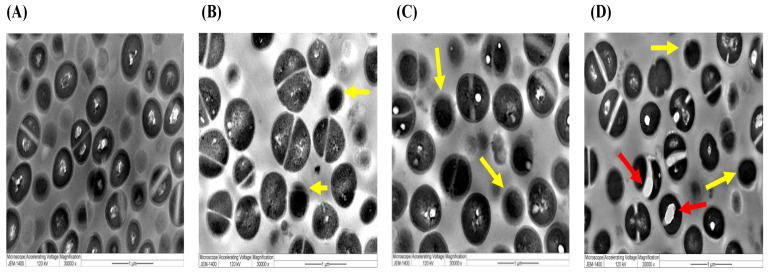
TEM images of *S. aureus*. The images showed the ultrastructural changes induced by the extracts of *A. sieberi* at the highest concentration (40 µg/mL) captured by the JEM-1400 transmission electron microscope at the magnification of 3000×. Red arrows indicate abnormal cellular vacuoles, while yellow arrows indicate undivided cells. (**A**) Control (DMSO), (**B**) aqueous extract, (**C**) ethanolic extract, and (**D**) *A. sieberi*-biosynthesized AgNPs.

As shown from the above data, *S. aureus* and *C. famata* were the most sensitive species to different *A. sieberi* treatments. The morphological and ultrastructural changes and their effect on the growth of the sensitive microbes were detected by TEM. As shown in Figure 11, the extracts of *A. sieberi* induced some morphological changes on the cells of *S. aureus* that looked more granulated and less proliferated compared to the control. In the AgNPs settings, more extensive ultrastructural changes occurred, where larger vacuoles were formed and the cellular division was terminated. 

**Figure 12:**
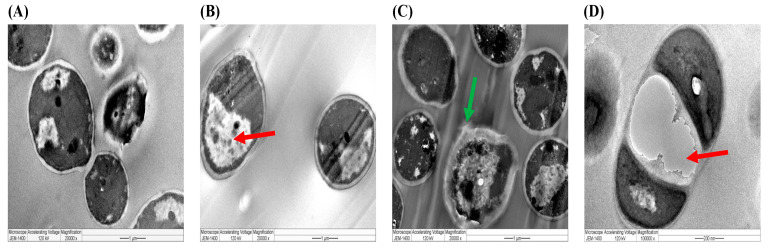
TEM images of *C. famata*. The images showed the ultrastructural changes induced by extracts of *A. sieberi* at the highest concentration (40 µg/mL) captured by the JEM-1400 microscope at the magnification of 2000×. Red arrows indicate abnormal cellular vacuoles, while the green arrow indicates raptured cellular membrane. (**A**) Control (DMSO), (**B**) aqueous extract, (**C**) ethanolic extract, and (**D**) *A. sieberi*-biosynthesized AgNPs (10000×).

Similarly, the mycelial growth of *C. famata* by different extracts of *A. sieberi* had serious morphological changes in the cellular structure. The aqueous extract induced the formation of large vacuoles, while the ethanolic extract induced extreme damage, as indicated by the rapture of the fungal cells, which further affected the mycelial growth (Figure 12). Larger vacuoles and undivided cells were induced by AgNPs.

## 3. Discussion

Medicinal plants are traditionally used worldwide as remedies for various diseases, including asthma, gastrointestinal symptoms, skin disorders, respiratory and urinary problems, and hepatic and cardiovascular diseases [22]. The current study aimed to investigate the antimicrobial activity of *A. sieberi* extracts and the biosynthesized AgNPs against selected strains of human pathogenic bacteria and fungi.

The phytochemical analysis of *A. sieberi* revealed that it was rich in amines, carboxylic groups (such as alcohols and phenols), and alkenes. In agreement with these findings, a previous study showed that the FTIR analysis of the leaf extract of *A. sieberi* had the highest peaks for hydroxyl groups, alkanes, alkenes, amides, and amines [23,24]. In addition, the study conducted by Alotibi and Rizwana (2019) revealed that the methanolic extract of *A. sieberi* was rich in the functional groups of OH, CH stretching, C꞊O stretching, and aromatic skeletal stretches, which agreed with the current results as well [25]. Other species of the Asteraceae family had a similar composition of alkyl halides, alkanes, alkenes, aldehydes, and amide groups, such as *Matricaria chamomilla* [26], *Centaurea cyanus* [27], *Artemisia maritima* [28], *Artemisia indica*, and *Artemisia vestita* [29]. 

In the current study, AgNPs produced a SPR peak of 445 nm compared to the crude extract, which did not reveal any absorption peaks. The appearance of an absorption peak at a wavelength of 400–500 nm is an indication that AgNPs were formed [30]. Similar studies reported the UV–visible spectrum of AgNPs biosynthesized from the extracts of *A. sieberi* at 482 nm [31], 407 nm [32], and 410 nm [33].

The secondary metabolites in the plant constituents are reliable resources for future free radical scavenging, anti-inflammatory activity, and antimicrobial agents. Plants are rich in terpenoids, phenolic compounds (flavonoids, phenolic acids, quinones, coumarins, lignans, stilbenes, and tannins), alkaloids, amines, betalains, and carotenoids [34,35]. Traditionally, *A. sieberi* has been used for its pharmaceutical and botanical importance and used previously to manage several diseases and disorders such as hepatocyte enlargement, hepatitis, gastritis, jaundice, wound healing, splenomegaly, dyspepsia, indigestion, flatulence, gastric pain, anemia, and anorexia [2]. In the current study, the GC-MS analysis resulted in many secondary metabolites, including 2-hexoxyethanol, chloral, dihexyl ether, dichloroacetic acid, piperidine, N, N-bis(2-hydroxyethyl)-2-aminoethanesulfonic acid, phenprobamate, cumene hydroperoxide, and 4-ethyl-o-xylene. A previous study analyzed the constituents of the essential oil of *A. sieberi* and reported that it contained monoterpenes (53.3%) (such as 1,8-Cineole, Artemisia ketone, and Nordavanone) and sesquiterpenes (37.3%) (such as β-caryophyllene, germacrene-d, and davanone) [36]. 

Previous studies confirmed that *Artemisia* spp. had many activities including antioxidant, antifungal, antimicrobial, anthelmintic, anti-ulcer, anticarcinogenic, hepatoprotective, neuroprotective, anti-depressant, analgesic, immunomodulatory, and cytotoxic effects [2,3,5,37,38]. Recently, it was revealed that the biological activity of *Artemisia* spp. has several other functions, such as antiprotozoal, antibacterial, antifungal, anti-ulcer, hepatoprotective, anti-inflammatory, immunomodulatory, cytotoxic, analgesic, neuroprotective, anti-depressant, precognitive, neurotrophic, cell membrane stabilizing, and antioxidant activities [6]. 

In the current study, we compared the antimicrobial activities of the crude aqueous and ethanolic extracts of *A. sieberi* against Gram-positive and Gram-negative bacteria and *Candida* fungal strains. In addition, we studied the antimicrobial properties of the biosynthesized AgNPs of *A. sieberi*. Significant activities were reported against the growth of Gram-positive bacteria *B. subtilis* and *S. aureus*. The Gram-positive bacteria showed a larger ZOI than the Gram-negative bacteria due to the variation in cell wall composition. Additionally, in human pathogenic fungal strains treated with AgNPs nanoparticles of the extracts of *A. sieberi* at different concentrations (10, 20, and 40 µg/mL), significant variability in the inhibition rates was reported against different fungal strains such as *C. albicans*, *C. parapsilosis*, *C. famata*, *C. krusei*, and *C. rhodotorula*. 

Previous studies showed that the significant arability and the selective activity of plant extracts towards the bacteria strains might be related to the presence of an impermeable barrier of lipopolysaccharide on the outer membrane of Gram-negative bacteria that inhibit diffusion of active compounds [39]. Moreover, Gram-positive bacteria freely allow the direct contact of active constituents with the phospholipid bilayer of the cell membrane, leading to enhanced ion permeability [40]. In agreement with our findings, the previous study of Alotibi and Rizwana (2019) [25] revealed that the methanol and dichloromethane extracts from *A. sieberi* had significant inhibitory effects against the growth of *Alternaria alternata*, *Fusarium moniliforme*, *F. solani*, *F. oxysporum*, and *Macrophomina phaseolina*. Another study showed that 8R-dihydroxygermacr-4(15),9(10)-dien-6S,7S,11RH,12,6-olide, 1R, 8S-dihydroxy-11R,13-dihydrobalchanin, 11-epiartapshin, and 3′-hydroxygenkwanin isolated from *A. sieberi* had moderate antifungal activities against *C. albicans*, *C. tropicalis*, *Aspergillus niger*, *F. solani*, and *A. alternata* [41]. The study conducted by Ghasemi et al. (2021) suggested that the strong antifungal activity of *A. sieberi* against *Botrytis cinerea* occurs due to the monoterpene compounds in the essential oils of *A. sieberi* [42]. Another study revealed that the essential oils extracted from Saudi-native species *of Artemisia absinthium*, *Artemisia scoparia*, and *A. sieberi* showed significant inhibitory activities against the growth of Gram-positive (*S. aureus*, *Bacillus licheniformis*, and *Micrococcus luteus*) and Gram-negative bacteria (*Enterobacter xiangfangensis*, *Escherichia fergusonii*, and *P. aeruginosa*) and yeast strains (*C. albicans*, *C. parapsilosis*, and *Aspergillus parasiticus*) with MIC values of 0.1–3 μg/mL [3]. In addition, in the study conducted by Mahboubi et al. (2015), the essential oils of *A. sieberi* had significant antimicrobial activities against *S. aureus* (MIC: 6.25–9.37 μL/mL), *C. albicans*, *P. aeruginosa*, and *E. coli* (MIC: 12–19 μL/mL) [43].

The antimicrobial activity of synthesized AgNPs depends mainly on the physical and chemical characteristics of the green synthesized particles, and plants use spherical shapes and small sizes of nanoparticles, which are more effective than the triangular ones [44,45]. However, the exact mechanism of how shape affects the antibacterial activity is still unclear. In addition, the antibacterial activity increases with AgNPs concentration and exposure time to microbes such as Gram-negative and Gram-positive microorganisms [46,47]. Similarly, our findings demonstrated that antibacterial and antifungal activity against some drug-resistant pathogenic strains was maximal at the highest concentrations of AgNPs. Additionally, more studies explained the mechanism of AgNPs impact on microbial cells via intracellular physicochemical processes, particularly the oxidation of protoplasm and its destruction by oxygen; AgNPs, in turn, play the role of catalysts [48]. In addition, other studies have proposed that the effect of AgNPs on membrane permeability and membrane-associated enzymes, including the proton FOF1-ATPase, can be the main mechanism of AgNPs [49]. Moreover, the complexation of cellular nucleic acid with heavy metals in AgNPs particles leads to DNA impairment and bacterial viability [50]. Other studies revealed the significant antimicrobial activities of AgNPs biosynthesized from culture supernatant of bacterial strains *Pseudoduganella eburnea* against *P. aeruginosa* (MIC: 6.25 μg/mL) and *S. aureus* (MIC: 100 μg/mL) [51], *Paenarthrobacter nicotinovorans* against *P. aeruginosa* (MIC: 25 μg/mL) and *Bacillus cereus* (MIC: 12.5 μg/mL) [52], and *Sphingobium* sp. against *E. coli* (MIC: 6.25 μg/mL) and *S. aureus* (MIC: 50 μg/mL) [53], which are drug-resistant pathogenic microbes. In general, it was proposed that AgNPs might be adsorbed on the cell membrane, therefore obstructing the growth of bacteria and having a protective function [33,54]. 

Recently, it was reported that the green synthesis of AgNPs from *Artemisia* extracts has a versatile range of biological applications and can be used as an eco-friendly material without harmful effects on the environment [33]. In the current study, the results suggested that the biosynthesized AgNPs using *A. sieberi* extract had a higher antimicrobial activity which significantly increased with higher concentrations of biogenic AgNPs. Thus, it could be used as an excellent source against the tested microbes. However, the microbicidal effect of biosynthesized AgNPs could be different depending on the organism tested. 

The results revealed more reliable antimicrobial activity of AgNPs than that of the crude extracts of *A. sieberi* against all bacterial and *Candida* species. However, the inhibitory effects induced by the plant extracts were higher than those of the AgNPs at different doses. The high biocidal activity of silver nanoparticles is explained by their large surface area, which provides better contact with microorganisms. Moreover, silver nanoparticles act as reservoirs for the AgNPs bactericidal agent. Previously, it was reported that a combination of silver nanoparticles and herbal extracts, their metabolites, and antibiotics exhibited a synergistic effect [55].

TEM microscopic analysis showed that different concentrations of aqueous and ethanolic extracts of *A. sieberi* significantly bind with cellular organelles and compartments of *S. aureus* cell membranes. *S. aureus* cultured in a control medium displayed some conserved morphological characteristics as intact cells. The treatment with the extract at different concentrations facilitated the penetration of the cell membranes of *S. aureus*, which led to the disruption of the cell wall and an increase in vacuole size with dilatation of the cell wall and lysis with increasing the time of exposure. The treatment with AgNPs at different concentrations induced the disintegration of bacterial cells, disruption of the cell walls and cell membranes, separation of the cytoplasmic membrane from the cell wall, and cytoplasmic dissolution, which significantly increased at higher concentrations of AgNPs.

Similarly, the ultrastructural changes in *C. famata* were identified by TEM analysis. It revealed a typical cellular structure with a conserved and intact layered cell wall and distinctive cytoplasmic membranes in the untreated *C. famata*. In contrast, fungal cells exposed to crude extracts of *A. sieberi* lost their cell wall permeability. In cells treated with AgNPs, disruption of the outer cell walls causes, subsequently, more permeabilization, which allows smaller AgNPs get inside the yeast. In consequence, that led to cellular vacuolation, destruction, and more accumulation of nanoparticles, which increased disruption, cellular vacuoles, aggregation, and dilatation of the cell walls, which more precisely affected the survival of both the yeast and the filamentous forms of the fungus compared to that which occurs in cells treated with pure aqueous and ethanolic extracts. The effect of AgNPs significantly occurred in a dose-dependent manner, which consequently increased with high doses. 

TEM images revealed that AgNPs formed or were deposited on the cell membrane of bacterial or fungal species, whereas various magnifications of AgNPs-treated microbes (bacteria and fungi) showed several black spots on the cellular membrane and cytoplasm. Based on ultrastructural analyses, it was proposed that the antimicrobial activity of the synthesized AgNPs of *A. sieberi* depends mainly on the physical interactions between the AgNPs and the microbial cell surface. That depends on the superior surface area of the particles of the Artemisia extracts which increase the long-term stability of AgNPs and affect cell wall permeability [56]. The antibacterial efficacy of AgNPs from *A. sieberi* against Gram-positive bacteria was better than that of Gram-negative bacteria due to the difference in biological activity and thickness of the cell walls of individual bacterial species. 

These results proved that AgNPs were mostly synthesized in the outer membrane of the cells of targeted microbes [57]. Previous studies on chitosan alone or as AgNPs proved antibacterial activity against *S. aureus* and *E. coli*. TEM analysis showed that chitosan nanoparticles induced cellular ultrastructure changes such as collapsed cell walls, condensed chromatin, and an increase in intracellular structures, such as vacuoles and cell debris, minimizing the growth and increasing the mortality score of the bacteria [58]. In agreement with our findings, the TEM results of the study conducted by Al-Otaibi and Rizwana (2019) [25] showed that damage to the fungal cells treated with an ethanolic extract of *A. sieberi* was represented by heavy vacuolation, proliferation of lipid bodies, septal damage, and undeveloped conidia.

In general, past observations supported the finding that that AgNPs nanoparticles alone or fabricated with plant extracts proved that nanoparticles were highly toxic and caused changes in the morphology and dimensions of the target organisms studied [59,60]. It was reported previously that increasing the concentration of AgNPs leads to a reduction in cell growth and more inhibition, which might reach 98.7% at higher concentrations. In addition, images of SEM and TEM analysis reflected the effect of nanoparticles via a shrinking and damaging of cell walls, indicating the toxicity of silver nanoparticles toward microorganisms such as cyanobacteria [60]. Similarly, previous studies proved that nanoparticles could affect bacterial replication, protein leakage, and cell death. That might be a potentially effective agent against the propagation and overgrowth of chronic microbial infections [57].

## 4. Materials and Methods

### 4.1. Plant Material and Preparation of Extracts

*A. sieberi* plant material was collected in March 2019 from the northern region (Rafha region), Kingdom of Saudi Arabia. The aerial parts (shoot) from each plant were washed, dried in shade, finely powdered, and stored at 4 °C for further use.

For the preparation of extracts, different solvents (ethanol and water) were used. A total of 30 g of the fresh, dry plant material was socked in 300 mL of either distilled water (aqueous extract) or absolute ethanol (ethanolic extract) for 24 h at room temperature. The mixture was transferred to a rotary shaker for another night, then filtered through cotton and tissue papers (Sartolab^®^ RF Vacuum Filtration Units 180C8, Goettingen, Germany). Finally, the remaining filtrates were left to dry in cleaned metallic trays, collected in clean glass containers, and stored at 4 °C. All plant extract (ethanol, water) concentrations were prepared as grams per milliliter. We used <1% concentrations combined with the extracts in culture media to increase their solubility. DMSO is widely used to solubilize different therapeutic applications, and studies indicated that a 10% *v/v* concentration did not modify culture viability [61]. Each stock-sterile-filtered extract was mixed with either the Mueller–Hinton agar medium for bacteria or the Sabouraud agar (Sigma-Aldrich, St. Louis. MO, USA) for *Candida* sp. to obtain different concentrations (0, 10, 20, and 40 µg/mL). 

### 4.2. Biosynthesis and Characterization of Silver Nanoparticles

The prepared extract of *A. sieberi* (2 g) was soaked in 10 mL of sterile water at 50 °C for 20 min, also filtered. The biosynthesis of silver nanoparticles was carried out by mixing 2 mL of *A. sieberi* extract with 100 mL of AgNO_3_ (2 mM). The set admixture was left unperturbed under the sun till the color of the response admixture changed to sanguine-brown. The color change is the primary suggestion of the conflation of the AgNPs, as shown by Kumar et al. (2015) with variations [62]. 

TEM imaging was used to confirm the liquid structure of the synthesized nanoparticles. The medication and processing of slides were performed according to the manufacturer’s instructions, as described preliminarily [63]. The samples were fixed by overnight buffering with glutaraldehyde at 2.5%, then washed and dehydrated by immersion in serial concentrations of ethanol. These samples were embedded by resin mixture and cut at thicknesses of 70–80 nm by a UC6 ultramicrotome (Leica, Wetzlar, Germany). After loading on the cupper grid, the slides were stained by uranyl acetate and lead citrate and analyzed by the JEM-1400 transmission electron microscope (JEOL Ltd. Inc., Tokyo, Japan).

The synthesized nanoparticles were purified by the PD-10 desalting column (Sigma-Aldrich, St. Louis, MI, USA) according to the manufacturer’s instructions [63]. 

A UV–visible spectrophotometer (Shimadzu, Tokyo, Japan) was used for the characterization of the purified biosynthesized AgNPs at 200–800 nm, according to the manufacturer’s instructions [64,65]. 

To measure the stability of the colloidal nanoparticles and detect the particle size distribution, we used the DLS technique, which calculated the PDI and the Z-average. The Zetasizer Pro (Malvern Panalytical, Malvern, UK) was used according to the manufacturer’s instructions.

### 4.3. Microbial Strains 

Four bacterial strains were kindly provided by the King Khalid University Hospital—Riyadh, Saudi Arabia: *Staphylococcus aureus* (ATCC-25923), *Bacillus subtilis* (ATCC-35021), *Escherichia coli* (ATCC-11775), and *Pseudomonas aeruginosa* (ATCC-27584) (ATCC, Manassas, VA, USA). All the bacterial strains were maintained on the Mueller–Hinton agar, then maintained on slants at 4 °C.

Five fungal strains were kindly provided by the Botany and Microbiology Department, King Saud University, Riyadh, KSA. *Candida parapsilosis* (ATCC-22019), *Candida krusei* (ATCC-14243), *Candida famata* (ATCC-36239), *Candida rhodotorula* (ATCC-66034), and *Candida albicans* (ATCC-60193). All the *Candida* strains were cultured on the Sabouraud agar and maintained on slants at 4 °C.

### 4.4. Gas Chromatography–Mass Spectrometry (GC-MS)

GC-MS was performed on the ethanol extract of *A. sieberi*. Helium gas with an inflow of 1 mL/min served as the carrier gas. The instrument was GC coupled with MS (7890A, 5975C (Agilent Technologies, Santa Clara, CA, USA). A phenyl–methyl siloxane column was used (30 m × 250 μm × 0.25 μm). The ensuing conditions were maintained during the GC run time (90 min): volume (1 μL), temperature (280 °C; 250 °C), ion source, and resolve rate (201). Further, the temperature was held for 5 min at 40 °C; to begin with, it was also increased to 280 °C at 10 °C/min and at this temperature, it was maintained for another 5 min. An electron impact (70 eV) was generated for mass spectroscopy with a checkup range of 35 to 780 *m*/*z*. The mass digital library of the National Institute of Standards and Technology (NIST) was used for identification [66].

### 4.5. Fourier-Transfigure Infrared Spectroscopy (FTIR)

FTIR analysis was used to identify the important functional groups present in the ethanol extract. The spectrometer (Nicolet—6700, Thermo Fisher Scientific Inc., Waltham, MS, USA) enjoying a ray splitter and a sensor (DTGS) equipped with OMNIC software was used to collect and dissect the diapason in the checkup range of 500–4000 cm^−1^. The obtained IR spectra were used to interpret the functional groups present according to the guidelines of LibreTexts libraries https://chem.libretexts.org/ (Accessed on 31 December 2022) [67].

### 4.6. Growth Inhibition Assay

Petri dishes of the Mueller–Hinton agar bacterial medium were prepared, and the different bacterial strains were plotted. With a metal cork, three discs were made and filled with one of the concentrations of the prepared treatments. Two dishes were prepared for the negative control (DMSO, <1%/dish) and positive control (cephalexin, 5 µg/mL). The cultures were incubated for 24 h at 37 °C. After incubation, all areas of inhibition were measured in millimeters. All experiments were prepared in triplicate.

Petri dishes of Sabouraud agar were prepared, and the different fungal strains were plotted. With a metal cork, three discs were made and filled with one of the concentrations of the prepared treatments. Another dish was prepared for the positive control (terbinafine, 5 µg/mL). The cultures were incubated for 72 h at 37 °C. After incubation, all areas of inhibition were measured in millimeters. All experiments were prepared in triplicate. Minimum inhibitory concentrations (MIC) and the evaluation of the susceptibility/resistance of the tested strains to various treatments were detected according to the guidelines of Clinical and Laboratory Standards Institute (CLSI) available from https://clsi.org/standards/products/microbiology/documents/m100/ (Accessed on 2 February 2022). 

### 4.7. Determination of the Effect of Plant Extracts on the Morphology and Ultrastructure of the Organisms under Study

To prepare the bacterial and fungal samples for scanning electron microscopy (SEM), the selected bacteria and fungus were grown in the presence of a certain concentration of aqueous and ethanolic plant extracts, where both the sterilized extracts—the Mueller–Hinton agar and the Sabouraud agar medium—were mixed separately immediately before pouring into the sterilized Petri dishes. Control samples (extracts untreated) were prepared in the Mueller–Hinton agar and the Sabouraud agar medium plates only (plant extract-free). Then, the samples were inoculated with 10 µL of fresh subculture (old) of the selected bacterial and fungal strains and incubated at 28 °C for 24 h for bacterial isolation and for 48 h for *Candida* isolation. These treated and untreated plates were sent to the electron microscopy lab in the Central Laboratory at the Women Students’ Medical Studies and Sciences Sections, King Saud University. All the sample preparations for the electron microscopy photography were performed according to their procedure.

### 4.8. Statistical Analysis

The results were analyzed using the STATISTIX 10 software, one-way analysis of variance (ANOVA), and Sidak’s multiple comparisons tests. Mean values were separated on the basis of the least significant difference (LSD). The statistical significance was set at *p* < 0.05.

## 5. Conclusions

*Artemisia*’s active metabolites may represent one of many examples of power hidden in natural sources; they might become a promising new antimicrobial drug, hopefully developed and introduced to clinical use shortly. Thus, more clinical trials are needed to increase and support the use of these extracts in clinical-based trials. The current study has several implications for scientific research. *A. sieberi* extracts produced by nanotechnology, in addition to being used in the inhibition programs of antimicrobials, might be used by food inspectors and food safety organizations. The results suggested that the AgNPs biosynthesized using *A. sieberi* extracts had potential antimicrobial activity against pathogenic bacterial and fungal strains and nullified resistance behavior. The biogenic AgNPs activity linked with the particles of *A. sieberi* extracts led to an increase in antibacterial activity and could be used as an excellent source against tested microbes. Despite the proposed high antimicrobial activity of the *A. sieberi* extracts and related AgNPs, further studies of their biocidal effect on animal models are needed before the use of synthesized AgNPs as antimicrobial agents.

## Figures and Tables

**Table 1:** The functional group analysis of *A. sieberi* by FT-IR.

Material	Absorption (cm^−1^)	Appearance	Group	Compound Class
Aqueous extract	3428	Strong, Broad	O-H Stretching	Alcohol
	2970, 2929	Weak, Broad	N-H Stretching	Amine Salt
	1633	Medium	C=C Stretching	Alkene
	1416, 1348	Medium	O-H Bending	Alcohol
	1081	Medium	C-N Stretching	Amine
	946	Strong	C=C bending	Alkene
AgNPs	3804, 3742	Medium, sharp	O-H Stretching	Alcohol
	3439	Strong, Broad	O-H Stretching	Alcohol
	2101	Weak	CΞC stretching	Alkyne
	1868, 1738	Weak	C-H bending	Aromatic compound
	1641	Medium	C=C Stretching	Alkene
	1562, 1544, 1520	Strong	N-O stretching	Nitro compound
	1058	Strong	C-O stretching	Primary alcohol
	671	Strong	C=C bending	Alkene
	617, 421	Strong	C-X	Halogen compound

**Table 2:** Phenolic constituents of the ethanolic extract of *A. sieberi*.

Phenolic Compound	Chemical Structure	Formula	Molecular Weight	Peak Area (%)
2-Hexoxyethanol	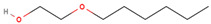	C_8_H_18_O_2_	148	30.5
Dihexyl ether	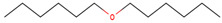	C_12_H_26_O	186	7.31
Dichloroacetic acid	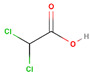	C_2_H_2_C_I2_O_2_	128	7.31
Piperidine	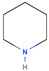	C_5_H_11_N	85	18.5
Chloral	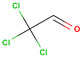	C_2_HCl_3_O	146	11.6
N, N-Bis(2-hydroxyethyl)-2-aminoethanesulfonic acid	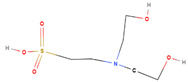	C_6_H_15_NO_5_S	213	9.34
Phenprobamate	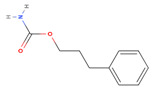	C_10_H_13_NO_2_	179	9.80
Cumene Hydroperoxide	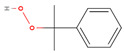	C_9_H_12_O_2_	152	7.70
4-Ethyl-o-xylene	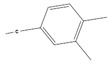	C_10_H_14_	134	4.97

**Table 3:** Antibacterial effects of *A. sieberi* (inhibition of growth).

Species		DMSO	AgNO_3_	Cephalexin	10 µg/mL	20 µg/mL	40 µg/mL	MIC
Aqueous Extract								
*E. coli*	Mean ± SD	0	-	18 ± 0 ^S^	0 ^R^	10 ± 0.5 ^R^	11.7 ± 0.3 ^R^	15 µg/mL ^R^
	Median (Min–Max)	0	-	18 (18–18)	0	10 (9.5–10.5)	11.5 (11.5–12)	
	*p* value	1		<0.001 *	1	<0.001 *	<0.001 *	
*B. subtilis*	Mean ± SD	0	-	25.8 ± 0.8 ^S^	9.7 ± 1.5 ^R^	10 ± 0.5 ^R^	13.2 ± 1.3 ^I^	<10 µg/mL ^R^
	Median (Min–Max)	0	-	26 (25–26.5)	10 (9.5–10.5)	10 (9.5–10.5)	13 (12–14.5)	
	*p* value	1		<0.001 *	<0.001 *	<0.001 *	<0.001 *	
*P. aeruginosa*	Mean ± SD	0	-	19.7 ± 0.6 ^S^	0 ^R^	0 ^R^	0 ^R^	>40 µg/mL ^R^
	Median (Min–Max)	0	-	20 (19–20)	0	0	0	
	*p* value	1		<0.001 *	1	1	1	
*S. aureus*	Mean ± SD	0	-	26.8 ± 0.8 ^S^	12 ± 1 ^I^	14.7 ± 1.5 ^I^	16.7 ± 0.6 ^I^	<10 µg/mL ^I^
	Median (Min–Max)	0	-	27 (26–27.5)	12 (11–13)	15 (13–16)	17 (16–17)	
	*p* value	1		<0.001 *	<0.001 *	<0.001 *	<0.001 *	
Ethanolic extract								
*E. coli*	Mean ± SD	0	-	18 ± 0 ^S^	0 ^R^	8.3 ± 0.6 ^R^	10.2 ± 0.3 ^R^	15 µg/mL ^R^
	Median (Min–Max)	0	-	18 (18–18)	0	8 (8–9)	10 (10–10.5)	
	*p* value	1		<0.001 *	1	<0.001 *	<0.001 *	
*B. subtilis*	Mean ± SD	0	-	25.8 ± 0.8 ^S^	12.3 ± 0.8 ^I^	14 ± 0 ^I^	16.5 ± 0.5 ^I^	<10 µg/mL ^I^
	Median (Min–Max)	0	-	26 (25–26.5)	12.5 (11.5–13)	14 (14)	16.5 (16–17)	
	*p* value	1		<0.001 *	<0.001 *	<0.001 *	<0.001 *	
*P. aeruginosa*	Mean ± SD	0	-	19.7 ± 0.6 ^S^	0 ^R^	0 ^R^	0 ^R^	>40 µg/mL ^R^
	Median (Min–Max)	0	-	20 (19–20)	0	0	0	
	*p* value	1		<0.001 *	1	1	1	
*S. aureus*	Mean ± SD	0	-	26.8 ± 0.8 ^S^	9.7 ± 0.3 ^R^	13.8 ± 0.8 ^I^	18 ± 0.5 ^I^	<10 µg/mL ^I^
	Median (Min–Max)	0	-	27 (26–27.5)	9.5 (9.5–10)	14 (13–14)	18 (17.5–18.5)	
	*p* value	1		<0.001 *	<0.001 *	<0.001 *	<0.001 *	
*A. sieberi* AgNPs								
*E. coli*	Mean ± SD	0	7.7 ± 0.6 ^R^	18 ± 0 ^5^	12 ± 0 ^R^	13.7 ± 0.3 ^I^	14.8 ± 0.3 ^I^	<10 µg/mL ^I^
	Median (Min–Max)	0	8 (7–8)	18 (18–18)	12 (12)	13.5 (13.5–14)	15 (14.5–15)	
	*p* value	1	<0.001 *	<0.001 *	<0.001 *	<0.001 *	<0.001 *	
*B. subtilis*	Mean ± SD	0	7.3 ± 0.3 ^R^	25.8 ± 0.8 ^S^	11 ± 0 ^R^	12.7 ± 0.6 ^I^	14 ± 0.5 ^I^	<10 µg/mL ^I^
	Median (Min–Max)	0	7.5 (7–7.5)	26 (25–26.5)	11 (11)	13 (12–13)	14 (13.5–14.5)	
	*p* value	1	<0.001 *	<0.001 *	<0.001 *	<0.001 *	<0.001 *	
*P. aeruginosa*	Mean ± SD	0	8.2 ± 0.6 ^R^	19.7 ± 0.6 ^S^	11.7 ± 0.3 ^R^	15 ± 1 ^I^	17 ± 0 ^I^	<10 µg/mL ^I^
	Median (Min–Max)	0	8.5 (7.5–8.5)	20 (19–20)	11.5 (11.5–12)	15 (14–16)	17 (17)	
	*p* value	1	<0.001 *	<0.001 *	<0.001 *	<0.001 *	<0.001 *	
*S. aureus*	Mean ± SD	0	8.7 ± 0.6 ^R^	26.8 ± 0.8 ^S^	12 ± 0 ^R^	14.7 ± 0.6 ^I^	19 ± 1 ^I^	<10 µg/mL ^I^
	Median (Min–Max)	0	9 (8.5–9)	27 (26–27.5)	12 (12)	15 (14–15)	19 (18–20)	
	*p* value	1	<0.001 *	<0.001 *	<0.001 *	<0.001 *	<0.001 *	
Summarized comparative data analysis (mean ± SD) of the highest effect at the highest dose (40 µg/mL).								
Organisms	Aqueous extract	Ethanolic extract	AgNPs	*p*-value				
				Aqueous vs. Ethanolic	Aqueous vs. AgNPs		Ethanolic vs. AgNPs	
*E. coli*	11.7 ± 0.3	10.2 ± 0.3	14.8 ± 0.3	0.28	0.77		0.74	
*B. subtilis*	13.2 ± 1.3	16.5 ± 0.5	14 ± 0.5	0.75	0.27		0.04 *	
*P. aeruginosa*	0	0	17 ± 0	-	<0.001 *		<0.001 *	
*S. aureus*	16.7 ± 0.6	18 ± 0.5	19 ± 1	0.85	0.61		0.92	

* Significant when *p* < 0.05, the results obtained by the chi-square test. ^S^: susceptible; ^R^: resistant; ^I^: intermediate.

**Table 4:** Antifungal effects of *A. sieberi* (inhibition of mycelial growth).

Species		DMSO	AgNO_3_	Terbinafine	10 µg/mL	20 µg/mL	40 µg/mL
Aqueous Extract							
*C. albicans*	Mean ± SD	0	-	41 ± 0	0	0	0
	Median (Min–Max)	0	-	41 (41)	0	0	0
	*p* value	1		<0.001 *	1	1	1
*C. famata*	Mean ± SD	0	-	38.8 ± 0.8	12.5 ± 0.5	13.3 ± 0.6	14.8 ± 0.3
	Median (Min–Max)	0	-	39 (38–39.5)	12.5 (12–13)	13 (13–14)	15 (14.5–15)
	*p* value	1		<0.001 *	<0.001 *	<0.001 *	<0.001 *
*C. krusei*	Mean ± SD	0	-	42 ± 1	6.3 ± 0.6	8.7 ± 0.6	10 ± 0
	Median (Min–Max)	0	-	42 (41–43)	6 (6–7)	9 (8–9)	10 (10)
	*p* value	1		<0.001 *	<0.001 *	<0.001 *	<0.001 *
*C. parapsilosis*	Mean ± SD	0	-	29.2 ± 0.3	0	0	0
	Median (Min–Max)	0	-	29 (29–29.5)	0	0	0
	*p* value	1		<0.001 *	1	1	1
*C. rhodotorula*	Mean ± SD	0	-	21.7 ± 0.6	0	0	0
	Median (Min–Max)	0	-	22 (21–22)	0	0	0
	*p* value	1		<0.001 *	1	1	1
Ethanolic extract							
*C. albicans*	Mean ± SD	0	-	41 ± 0	0	0	0
	Median (Min–Max)	0	-	41 (41)	0	0	0
	*p* value	1		<0.001 *	1	1	1
*C. famata*	Mean ± SD	0	-	38.8 ± 0.8	25.5 ± 0.5	29.8 ± 0.8	35.8 ± 0.3
	Median (Min–Max)	0	-	39 (38–39.5)	25.5 (25–26)	30 (29–30.5)	36 (35.5–36)
	*p* value	1		<0.001 *	<0.001 *	<0.001 *	<0.001 *
*C. krusei*	Mean ± SD	0	-	42 ± 1	0	0	0
	Median (Min–Max)	0	-	42 (41–43)	0	0	0
	*p* value	1		<0.001 *	1	1	1
*C. parapsilosis*	Mean ± SD	0	-	29.2 ± 0.3	9.3 ± 0.3	11.3 ± 0.3	18.8 ± 0.3
	Median (Min–Max)	0	-	29 (29–29.5)	9.5 (9–9.5)	11.5 (11–11.5)	19 (18.5–19)
	*p* value	1		<0.001 *	<0.001 *	<0.001 *	<0.001 *
*C. rhodotorula*	Mean ± SD	0	-	21.7 ± 0.6	0	0	0
	Median (Min–Max)	0	-	22 (21–22)	0	0	0
	*p* value	1		<0.001 *	1	1	1
*A. sieberi* AgNPs							
*C. albicans*	Mean ± SD	0	10 ± 0	41 ± 0	11 ± 0	12 ± 0	14.3 ± 0.8
	Median (Min–Max)	0	10 (10)	41 (41)	11 (11)	12 (12)	13.5 (13.5–15)
	*p* value	1	>0.05	<0.001 *	<0.001 *	<0.001 *	<0.001 *
*C. famata*	Mean ± SD	0	23.5 ± 0	38.8 ± 0.8	22.3 ± 0.6	24.7 ± 0.3	26.2 ± 0.8
	Median (Min–Max)	0	13.5 (13.5)	39 (38–39.5)	22 (22–23)	24.5 (24.5–25)	26 (25.5–27)
	*p* value	1	>0.05	<0.001 *	<0.001 *	<0.001 *	<0.001 *
*C. krusei*	Mean ± SD	0	10.5 ± 0	42 ± 1	10.7 ± 0.6	12 ± 0	14.7 ± 0.8
	Median (Min–Max)	0	10.5 (10.5)	42 (41–43)	11 (10–11)	12 (12)	14 (13.5–15)
	*p* value	1	>0.05	<0.001 *	<0.001 *	<0.001 *	<0.001 *
*C. parapsilosis*	Mean ± SD	0	6 ± 0	29.2 ± 0.3	6.3 ± 0.6	7 ± 0	8.3 ± 0.6
	Median (Min–Max)	0	6 (6)	29 (29–29.5)	6 (6–7)	7 (7)	8 (8–9)
	*p* value	1	>0.05	<0.001 *	<0.001 *	<0.001 *	<0.001 *
*C. rhodotorula*	Mean ± SD	0	8 ± 0	21.7 ± 0.6	7 ± 0	8.8 ± 0.3	10.3 ± 0.6
	Median (Min–Max)	0	8 (8)	22 (21–22)	7 (7)	9 (8.5–9)	10 (10–11)
	*p* value	1	>0.05	<0.001 *	<0.001 *	<0.001 *	<0.001 *
Summarized comparative data analysis (mean ± SD) of the highest effect at the highest dose (40 µg/mL).							
Organisms		Aqueous extract	Ethanolic extract	AgNPs	*p*-value		
					Aqueous vs. Ethanolic	Aqueous vs. AgNPs	Ethanolic vs. AgNPs
*C. albicans*		0	0	14.3 ± 0.8	-	-	0.29
*C. famata*		14.8 ± 0.3	35.8 ± 0.3	26.2 ± 0.8	<0.001 *	<0.001 *	-
*C. krusei*		10 ± 0	0	14.7 ± 0.8	<0.001 *	<0.001 *	<0.001 *
*C. parapsilosis*		0	18.8 ± 0.3	8.3 ± 0.6	-	-	<0.001 *
*C. rhodotorula*		0	0	10.3 ± 0.6	-	-	-

* Significant when *p* < 0.05, the results obtained by the chi-square test.

## Data Availability

Not applicable.
